# A General Protein Unfolding‐Chemical Coupling Strategy for Pure Protein Hydrogels with Mechanically Strong and Multifunctional Properties

**DOI:** 10.1002/advs.202102557

**Published:** 2021-12-22

**Authors:** Ziqing Tang, Huacheng He, Lin Zhu, Zhuangzhuang Liu, Jia Yang, Gang Qin, Jiang Wu, Yijing Tang, Dong Zhang, Qiang Chen, Jie Zheng

**Affiliations:** ^1^ Wenzhou Institute University of Chinese Academy of Sciences Wenzhou 325001 China; ^2^ College of Chemistry and Materials Engineering Wenzhou University Wenzhou Zhejiang 325035 China; ^3^ Oujiang Laboratory (Zhejiang Lab for Regenerative Medicine, Vision and Brain Health) Wenzhou Zhejiang 325000 China; ^4^ School of Materials Science and Engineering Henan Polytechnic University Jiaozuo 454003 China; ^5^ School of Pharmaceutical Sciences Key Laboratory of Biotechnology and Pharmaceutical Engineering Wenzhou Medical University Wenzhou Zhejiang 325035 China; ^6^ Department of Chemical, Biomolecular, and Corrosion Engineering The University of Akron Akron OH 44325 USA; ^7^ Wenzhou Key Laboratory of Perioperative Medicine The First Affiliated Hospital of Wenzhou Medical University Wenzhou Zhejiang 325000 China

**Keywords:** hydrogel sensors, mechanical strength, protein hydrogels, protein unfolding

## Abstract

Protein‐based hydrogels have attracted great attention due to their excellent biocompatible properties, but often suffer from weak mechanical strength. Conventional strengthening strategies for protein‐based hydrogels are to introduce nanoparticles or synthetic polymers for improving their mechanical strength, but often compromise their biocompatibility. Here, a new, general, protein unfolding‐chemical coupling (PNC) strategy is developed to fabricate pure protein hydrogels without any additives to achieve both high mechanical strength and excellent cell biocompatibility. This PNC strategy combines thermal‐induced protein unfolding/gelation to form a physically‐crosslinked network and a ‐NH2/‐COOH coupling reaction to generate a chemicallycrosslinked network. Using bovine serum albumin (BSA) as a globular protein, PNC‐BSA hydrogels show macroscopic transparency, high stability, high mechanical properties (compressive/tensile strength of 115/0.43 MPa), fast stiffness/toughness recovery of 85%/91% at room temperature, good fatigue resistance, and low cell cytotoxicity and red blood cell hemolysis. More importantly, the PNC strategy can be not only generally applied to silk fibroin, ovalbumin, and milk albumin protein to form different, high strength protein hydrogels, but also modified with PEDOT/PSS nanoparticles as strain sensors and fluorescent fillers as color sensors. This work demonstrates a new, universal, PNC method to prepare high strength, multi‐functional, pure protein hydrogels beyond a few available today.

## Graphical abstract

A new, robust, and universal, crosslinking strategy is proposed and demonstrated to prepare high strength, multi‐functional, pure protein hydrogels without any synthetic components. Such general crosslinking property makes use of general heat‐induced protein unfolding and ‐NH2/‐COOH coupling to produce pure protein hydrogels, beyond a few available today.

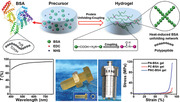

## 1 Introduction

Proteins as intricate macromolecules must fold into very delicate and precise conformations to achieve their biological activities. Due to their unique folding structures, functions, and chemistries, native proteins are often considered as biocompatible building blocks for developing new protein‐based biomaterials (e.g., hydrogels, tissue scaffolds, and polymer films) for many applications of tissue engineering,^[^ ^1^, ^2^, ^3^, ^4^, ^5^, ^6^ ^]^ tissue repair,^[^ ^7^, ^8^, ^9^, ^10^, ^11^ ^]^ drug delivery.^[^ ^12^, ^13^, ^14^, ^15^, ^16^ ^]^ Among them, protein‐based hydrogels offer a vast design space for generating a variety of multifunctional materials by engineering the sequences, structures, surface chemistry of proteins, and their interactions with hosting or counterpart polymers. Existing protein‐based hydrogels, despite less explored, can be generally categorized into two groups, that is, fibrillar protein‐ and globulin protein‐based hydrogels, in which the former hydrogels were formed by silk fibroin (SF), collagen, and elastin, while the latter ones by bovine serum albumin (BSA), lysozyme, and casein.^[^ ^17^, ^18^, ^19^, ^20^, ^21^ ^]^ Similar to conventional hydrogels, protein hydrogels can be physically, chemically, or hybrid crosslinked.^[^ ^22^, ^23^, ^24^, ^25^, ^26^, ^27^ ^]^ However, a long‐standing challenge for protein‐based hydrogels is how to reliably transform protein structures and functions into targeted polymers. Additional major concern includes weak mechanical properties, which greatly limit their applications requiring high mechanical properties.^[^ ^28^, ^29^, ^30^, ^31^, ^32^ ^]^

Different strategies have been developed to improve the mechanical properties of protein‐based hydrogels by the careful control of network structures and energy dissipation modes. A number of SF‐based double‐network (DN) and triple‐network (TN) hydrogels with physical, chemical, or hybrid crosslinkings exhibited extremely high compressive strength of 14–240 MPa.^[^ ^33^, ^34^, ^35^ ^]^ Gelatin hydrogels after soaking in a (NH_4_)_2_SO_4_ solution can also achieve a compressive stress of 12 MPa.^[^ ^36^ ^]^ In parallel to the abovementioned fibrillar protein‐based hydrogels, globulin protein hydrogels including egg white‐based hydrogels^[^ ^37^ ^]^ and BSA‐based DN hydrogels^[^ ^38^ ^]^ can also achieve very high compressive strength of 35–50 MPa. Of note, current protein‐based hydrogels, apart from globulin proteins, are generally composed of other network components, including synthetic polymers and ionic surfactants. Such hybrid nature may introduce potential problems in biocompatibility and biodegradability. To our knowledge, it is surprising that there are no any pure protein hydrogels being reported to date with high strength compared to articular cartilage, presumably due to lack of a new design concept and synthesis strategy to realize such gels. From a design viewpoint, it is also highly desirable to develop pure globulin protein hydrogels (PNGP gels) without any synthetic network components.

In this work, we developed a general and robust protein unfolding‐chemical coupling (PNC) strategy, a combination of a heat‐induced unfolding of globulin proteins and a chemical coupling reaction of 1‐(3‐Dimethylaminopropyl)‐3‐ethylcarbodiimide hydrochloride (EDC)/*N*‐Hydroxysuccinimide (NHS) between ‐NH_2_ and ‐COOH, to fabricate pure protein gels without any synthetic additives and polymers. BSA of 582 amino acids, as a typical natural globulin protein being abundantly presented in blood plasma, is widely used for different bio‐purposes and contains multifunctional groups and domains.^[^ ^39^, ^40^, ^41^ ^]^ Due to the intrinsic heat‐induced denaturation of any globulin protein,^[^ ^42^ ^]^ we developed a simple, one‐pot process to form pure BSA hydrogels with hybrid chemical and physical crosslinkings via a rapid, mild heating treatment within ≈1–10 min, allowing for the simultaneous unfolding‐gelation of BSA proteins and self‐coupling reactions of EDC/NHS between –NH_2_ and –COOH groups on BSA proteins.^[^ ^43^, ^44^ ^]^ The resultant PNC‐BSA gels were highly transparent and free‐shapeable. Under optimal conditions, PNC‐BSA gels achieved compressive strength of ≈115 MPa, which was far superior to that of protein unfolding‐gelation BSA gel (PN‐BSA gel, ≈4 MPa) as only prepared by heating treatment and protein coupling BSA gel (PC‐BSA gel, ≈20 MPa) as solely crosslinked by EDC/NHS, confirming synergistic mechanical enhancement by both heating‐induced unfolding and EDC/NHS coupling. Mechanistically, the presence of hybrid crosslinkers also empowers PNC‐BSA gels to achieve fast self‐recovery and good fatigue‐resistance properties, while the pure protein nature affords BSA gels excellent cell viability and negative hemolysis. Practically, upon introduction of conductive Poly(3,4‐ethylenedioxythiophene)/Poly(styrene sulfonate) (PEDOT/PSS) nanoparticles and fluorescent fillers into PNC‐BSA gels, they can further function as strain sensors and fluorescent materials. From a preparation viewpoint, we developed a new strategy to fabricate pure protein hydrogel with highly mechanical properties via combination of protein unfolding‐gelation and ‐NH_2_/‐COOH coupling reaction. The preparation strategy, in fact, reflects the unique network structures of this type of hydrogels, particularly for natural proteins. To our knowledge, no any hydrogel has been reported to be prepared and toughened in a way by simultaneous unfolding‐gelation and ‐NH_2_/‐COOH associations groups in a single material. This work demonstrates a simple preparation strategy to fabricate fully protein hydrogels with high mechanical strength, benign cell compatibility, and fast self‐recovery, allowing to be further engineered into smart materials and devices with additional and desirable functions.

## 2 Experimental Section

### 2.1 Materials

BSA (98%) and bromophenol blue (BPB) were purchased from Sigma Aldrich Inc. Albumin from milk (MA), albumin from dried egg white (OVA), EDC (>98.0%), and NHS (>98.0%) were purchased from TCI (Shanghai) Inc. Guanidine hydrochloride (GdnHCl, 99.0%), urea (99%) and sodium dodecyl sulfate (SDS, 92.5–100.5%) were purchased from Aladdin (Shanghai) Inc. SF and PEDOT/PSS were prepared according to the previous work.^[^ ^35^, ^45^ ^]^ Graphene quantum dot (GQD) was kindly provided by Dr. Fengna Xi's group at Zhejiang Sci‐Tech University. All materials were used without further purification.

### 2.2 Hydrogel Preparation

PNC‐BSA gels were synthesized by one‐pot method. Briefly, BSA (1.5 g, 500 mg mL^−1^) was dissolved into H_2_O (3 mL) in a reactor. Then, EDC and NHS solution (90 µL, 6 wt% of BSA, the weight ratio of EDC/NHS = 1, and the stock solution of both EDC and NHS were 1 g mL^−1^) were added into BSA solution and stirred for 10 s. After that, the obtained precursor solution was immediately injected into a mold and heated at 80 °C for 10 min in a water bath. As a result, PNC‐BSA gels could be successfully prepared. PC‐BSA gels were synthesized by similar method without heating process. PN‐BSA gels were directly synthesized through heating process without adding EDC and NHS solutions. PCN‐BSA gels were synthesized by heating PC‐BSA gels. EDC‐BSA and NHS‐BSA gels were synthesized by a similar method of PNC‐BSA while only adding EDC or NHS solution, respectively. Other protein‐based hydrogels were prepared by a similar method except for the different concentrations of proteins. Specifically, the concentration of silk fibrin (SF), gelatin, ovalbumin (OVA), and milk albumin (MA) were 120, 200, 400, and 400 mg mL^−1^.

### 2.3 Mechanical Tests

Compressive and tensile tests were carried out with a commercial universal testing machine (WSM‐10KN) with a loading cell of 100 N. For compressive tests, the as‐prepared cylindrical gels with 8.5 mm diameter were cut into 10 mm height samples before test, and compressed at a rate of 5 mm min^−1^. The compressive strain (*ε*_c_) was calculated as *h*/*h*_0_, where *h* was the deformed height and *h*_0_ was the original height, the compressive stress (*σ*_c_) was calculated as *F*/*A*_0_, where *F* was the force applied on the gel and *A*_0_ was the original cross‐sectional area of the gel sample. For tensile tests, the as‐prepared gel sheets were cut into dumbbell‐shape (length of 25 mm, width of 4 mm, and thickness of 1 mm) before tests. The tensile strain (*ε*_t_) was calculated as Δ*l*/*l*_0_, where *l* was the deformed length and *l*_0_ was the original length, the tensile stress (*σ*_t_) was calculated as *F′*/*A*_0_′, where *F′* was the force applied on the gel and *A*_0_′ was the original cross‐sectional area of the gel sample. The tensile rate was 20 mm min^−1^. For adhesive tests, the as‐prepared BSA solution (BSA of 500 mg mL^−1^, EDC/BSA = 6 wt%, EDC/NHS = 1/1) were quickly injected between two sheets of cleaned porcine skins with a 2 mm spacer. After gelling for 30 min at room temperature, 180° peeling and lap shear tests were conducted to evaluate the adhesion energy and adhesion strength. The adhesion energy was calculated by 2F/*w*, where *F* was the steady peeling force and *w* was the width of the gel. The adhesion energy was calculated by *F*/*S*, where *F* was the maximum peeling force and *S* was the area of the gel adhered to porcine skin.

The hysteresis tests of gels were also conducted by the same machine. The cylindrical gel samples (diameter of 8.5 mm, height of 10 mm) were compressed by a loading‐unloading cycle to a different maximum compressive strain (*ε*_c,max_), with a loading‐unloading rate of 5 mm min^−1^. For recovery experiments, the samples were first compressed by a loading‐unloading cycle to achieve a maximum compressive strain (*ε*_c,max_ = 50%) and then the samples were sealed in a plastic bag and stored at various temperatures (room temperature, 40, 60, and 80 °C) for various times (0, 3, 5, 10, and 40 min). Then, the samples were taken out and cooled down at room temperature prior to tests. For fatigue tests, the gel was sustained tenth loading‐unloading cycles with a recovery time of 5 min between two loading cycles.

### 2.4 Hydrogel Characterization

#### 2.4.1 Scanning Electron Microscope (SEM)

Morphologies of hydrogel samples were obtained by a field emission SEM (Merlin Compact, Zeiss) at an acceleration voltage of 15 kV. All gel samples were frozen and fractured in liquid nitrogen and dried in a lyophilizer. Before tests, the samples were coated with a thin layer of gold.

#### 2.4.2 Fourier‐Transform Infrared Spectroscopy

The as prepared gel samples (film with 3 mm thickness) were first immersed into water for 3 days to remove the unreacted EDC/NHS molecules. Then the spectra were recorded by a Fourier‐transform infrared (FTIR) spectrometer (Spectrum 3, PerkinElmer) with attenuated total reflection technique in the range of 4000–400 cm^−1^.

#### 2.4.3 Rheology

Rheological tests were carried on a rheometer (MCR302, Anton Paar). The gelation time tests were performed at angular velocity of 10 rad s^−1^, amplitude of 0.1%. The frequency sweep tests were performed at strain of 0.1%, frequency range of 0.1–100 rad s^−1^, and at 25 °C. The amplitude sweep tests were performed at angular velocity of 10 rad s^−1^, amplitude range of 0.01–100%.

#### 2.4.4 Swelling

The as‐prepared gels were soaked in deionized water, 4 m GdnHCl, 8 m urea, and 20 mm SDS for 1 week to reach swelling equilibrium. Excess solution from surface of the gels was removed by filter paper before test.

#### 2.4.5 Electricity

The electrical performance of PEDOT/PSS@PNC‐BSA gels was tested by a DC resistance tester (TH2515A, Changzhou Tonghui Electronic Co., Ltd.)

#### 2.4.6 Fluorescence

The PNC‐BSA gels with different filler were irradiated under a UV lamp (365 nm, 8 W, UVL‐18, UVP) and different fluorescent colors can be observed.

#### 2.4.7 Biocompatibility

Cytotoxicity: CCK‐8 assay was conducted to evaluate the cytotoxicity of the PNC‐BSA gels. The hydrogels were sterilized by an UV light sterilization for 2 h, then the gels were immersed into 1 mL complete media for 7 d. After filtration, the leach liquor was obtained. L929 cells were seeded into 96‐well plate with a density of 8 × 10^3^ cells well^−1^ and incubated for 24 h with leach liquor (for control, cells were incubated for 24 h with complete media). After that, CWT‐8 solutions were added into the plate and incubated for another 24 h, then UV–vis spectrometer was conducted to measure the absorbance at 450 nm.

#### 2.4.8 Hemolysis Test

1 mL of whole blood was first added to a 1.5 mL Eppendorf tube which contained 150 µL citric acid, then the obtained solution was centrifuged at 3500 rpm for 10 min at 4 °C to isolate red blood cells (RBCs). After three times centrifugation, the obtained RBCs were diluted by 20 mL phosphate buffered saline (PBS) for further use. For the hemolysis test, 200 µL RBCs suspension was added to 800 µL hydrogel suspension (for negative control, RBCs suspension was dispersed in PBS; for positive control, RBCs suspension was dispersed in DI water). All the suspensions were centrifuged at 10 016 × *g* for 3 min after being incubated in a rocking shaker at 37 °C for 3 h. The absorbance of the released hemoglobin in the suspensions was measured at 540 nm by an UV–vis spectrometer, and the hemolysis ratio was calculated by the following formula:

(1) $$\begin{array}{ccc} & & \mathrm{Hemolysis}\;\mathrm{ratio}\;\left(\%\right)\\ & & =\left\{\frac{\mathrm{Suspension}s_{\mathrm{abs}}-\mathrm{Negative}\;\mathrm{contro}l_{\mathrm{abs}}}{\mathrm{Positive}\;\mathrm{contro}l_{\mathrm{abs}}-\mathrm{Negative}\;\mathrm{contro}l_{\mathrm{abs}}}\right\}\;\times \;100\% \end{array}$$

### 2.5 Statistical Analysis

Cytotoxicity experiments were conducted six independent biological replicates. Data were presented by mean ± SEM and statistical analysis was performed using GraphPad Prism 7.0. When comparing the means of control and PNC‐BSA gel, an unpaired *t* test was performed. A *p*‐value less than 0.05 was considered as statistically significant: ***p < 0.001.

## 3 Results and Discussion

### 3.1 Network Structure of PNC‐BSA Gels

**Figure 1:**
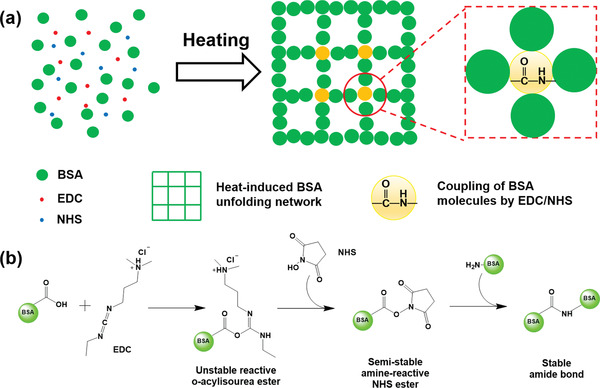
a) A one‐pot, protein unfolding‐chemical coupling (PNC) strategy for the preparation of PNC‐BSA hydrogels; b) EDC/NHS coupling reaction between carboxyl and amino groups of BSA molecules.

Inspired by a well‐known egg‐cooking phenomenon, PNC‐BSA hydrogels were prepared by a simultaneous combination of heat‐induced BSA unfolding‐gelation and EDC/NHS coupling reaction between BSA proteins. Briefly, as shown in **Figure** **1**a, once BSA was first dissolved in water at room temperature, EDC and NHS were immediately added to the BSA solution. Next, this precursor solution was heated up to 80 °C for inducing the unfolding, aggregation, and gelation of BSA proteins, resulting in a physical BSA network. Similar to the egg‐cooking process, the heat‐induced gelation of BSA has been well studied.^[^ ^42^, ^46^, ^47^ ^]^ Meanwhile, amide bonds were formed between ‐NH_2_ and ‐COOH groups of BSA molecules via a coupling reaction of EDC/NHS, leading to the secondary chemical crosslinking. Figure 1b shows the step‐by‐step coupling reaction pathways between BSA proteins. Specifically, carboxyl groups of BSA reacted with EDC, forming an unstable and amine‐reactive *o*‐acylisourea ester, which continuously reacted with NHS to form a semi‐stable amine‐reactive NHS ester. Then, NHS ester reacted with amino groups of other BSA to form an intermolecular amide bond, ending with a chemical coupling between two BSA proteins.^[^ ^48^, ^49^ ^]^ Ideally, since a single BSA protein contains 99 carboxyl groups and 60 amino groups,^[^ ^50^ ^]^ BSA proteins in solution can form a complex, chemically‐crosslinked network, complement to the denature‐induced physical network. This hypothesis was further proved by the FTIR spectra in Figure S1, Supporting Information. Two characteristic peaks of amide groups (1640 cm^−1^ of C═O stretching and 1544 cm^−1^ of N‐H bending) were shown in both PN‐BSA and PNC‐BSA gels while the relative peak of N‐H bending in PNC‐BSA gel was stronger than that of PN‐BSA gel, indicating more amide groups were formed. Here, we propose and demonstrate a simple, one‐pot method to prepare PNC‐BSA gels without any other synthetic additives and polymers via the heat‐induced unfolding‐gelation and the EDC/NHS coupling reactions.

### 3.2 Mechanical Properties of PNC‐BSA Gels

**Figure 2:**
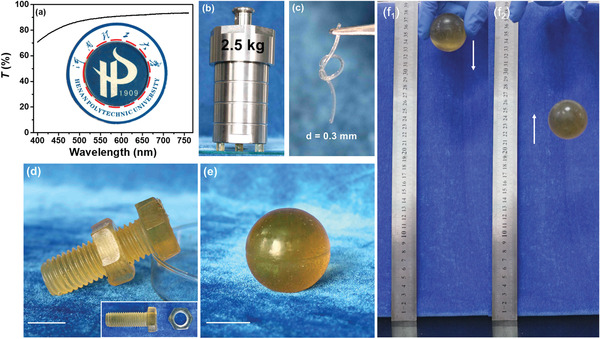
Visual inspection of PNC‐BSA hydrogels. a) Optical transmittance and transparency of PNC‐BSA gel. The gels can b) hold a 2.5 kg weight, c) be knotted, and be made into d) a well‐match pair of screw and bolt and e) a bouncing ball, f_1_,f_2_) which can be bounced from a ground by 67% in height. The scales in (d) and (e) are 2 cm.

At a first glance in **Figure** **2**a, as‐prepared PNC‐BSA gel demonstrated a high transparence with slightly light‐yellow appearance (as indicated by the red dotted circle) to clearly visualize the University Logo under the gel, and its optical transmission of the gel was up to 70% under visible light. Meanwhile, PNC‐BSA gels enabled to lift 2.5 kg of weight without being crushed (Figure 2b), and they also can be made into a knotted fiber with a diameter of 0.3 mm (Figure 2c), a well‐match hexagon screw, and bolt with very fine threads (Figure 2d), and a bouncing ball with excellent elasticity (Figure 2e,f). Qualitatively, these preliminary results demonstrated that our pure BSA gels exhibited both high mechanical strength and free‐shapeable property. Considering that the heat‐induced gelation of BSA gel occurred too rapid to be measured at 80 °C, we assessed the gelation time of PC‐BSA gel at room temperature by a rheometer to better understand its gelation process. As shown in Figure S2a, Supporting Information, both storage modulus (G′) and loss modulus (G′′) increased monotonically as time, during which G′ and G′′ were intersected at 8 min to indicate its gelation time. This also indicates that BSA is gelled rapidly in the presence of EDC/NHS even at room temperature. Amplitude sweep test in Figure S2b, Supporting Information showed that as amplitudes were less than 1%, both G′ and G′′ retained their values at stable plateau of ≈200 and 30 kPa, where G′ was always greater than G′′, indicating that PNC‐BSA hydrogel structure is stable at this amplitude range. Further frequency sweep test showed that i) G′ was always greater than G′′ in the range of 0.1–100 rad s^−1^ (Figure S2c, Supporting Information) and ii) both G′ and G′′ increased as *ω*, clearly indicating the strong viscoelasticity nature of PNC‐BSA gels.

**Figure 3:**
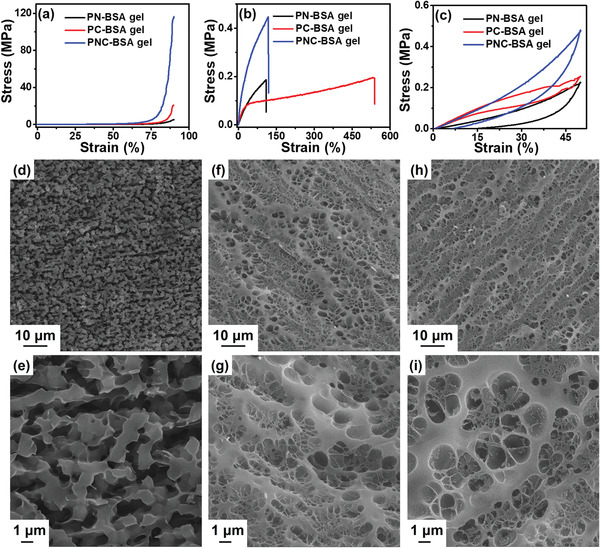
Side‐by‐side comparison of a) compressive curves, b) tensile curves, and c) compressive cycling curves between PN‐BSA, PC‐BSA, and PNC‐BSA gels. SEM images of d,e) PN‐BSA gel, f,g) PC‐BSA gel, and h,i) PNC‐BSA gel at different length scales.

We tested and compared the mechanical performance of three types of BSA gels, that is, PN‐BSA gels as prepared by heat‐induced unfolding‐gelation, PC‐BSA gels prepared by EDC/NHS coupling, and PNC‐BSA gels. As shown in **Figure** **3**a, PNC‐BSA gels significantly outperformed the other two BSA gels in terms of compressive properties. Specifically, compressive modulus (*E*_c_), compressive strain (*ε*_c,f_), and compressive stress (*σ*_c,f_) of PNC‐BSA gels were 971 kPa, 89.3%, and 115 MPa, respectively, in comparison with *E*_c_ of 74–946 kPa, *ε*_c,f_ of 84.3–89.3%, and *σ*_c,f_ of 4–51 MPa for the other two BSA gels (Figure 3a, Figure S3 and Table S1, Supporting Information). Side‐by‐side comparison of these mechanical data revealed several facts: i) NHS‐BSA hydrogel had similar *σ*_c,f_ to PN‐BSA gel, indicating that the introduction of NHS alone does not form chemical crosslinking of BSA. However, PNC‐BSA gels can achieve a compressive stress of 115 MPa, which was 2.7‐times stronger than that of EDC‐BSA gel (43 MPa), confirming that NHS increases the efficiency of EDC/NHS coupling; ii) The comparison of compressive properties between PC‐BSA gel and PN‐BSA gel showed that PC‐BSA gel (*σ*_c,f_ = 20 MPa) exhibited five times stronger than PN‐BSA gel (*σ*_c,f_ = 4 MPa), indicating that ‐NH_2_/‐COOH coupling of BSA has already obtained a very strong protein gel; iii) PNC‐BSA gel (115 MPa) was 5.8‐times stronger than PC‐BSA gel (20 MPa), indicating a synergistic effect of unfolding‐gelation and chemical coupling for achieving the extremely high strength of BSA gels; iv) PNC‐BSA gel was 2.2‐times stronger than PCN‐BSA gel (51 MPa), indicating that the one‐pot method is better than the two‐step process for fabricating high‐strength BSA gels.

In parallel to excellent compressive properties, Figure 3b consistently showed that PNC‐BSA gels also had the much higher tensile properties (*E*_t_ of 1 MPa, *σ*_t,f_ of 0.43 MPa, *ε*_t,f_ of 111%, and *W* of 0.31 MJ m^−3^) than PN‐BSA gels (*E*_t_ of 0.26 MPa, *σ*_t,f_ of 0.17 MPa, *ε*_t,f_ of 104%, and *W* of 0.11 MJ m^−3^) and PC‐BSA gels (*E*_t_ of 0.36 MPa, *σ*_t,f_ of 0.20 MPa, *ε*_t,f_ of 538%, and *W* of 0.71 MJ m^−3^). Cyclic loading‐unloading tests in Figure 3c showed that while PNC‐BSA, PN‐BSA, and PC‐BSA gels all exhibited hysteresis loops at *ε*_max_ of 50%, they dissipated different energies (*U*_hys_) of 38.47, 24.83, and 16.69 kJ m^−3^ respectively, clearly showing that the presence of unfolding‐coupling in BSA gels can effectively dissipate energy. In addition, as *ε*_max_ increased from 10 to 70%, *U*_hys_ of PNC‐BSA gels increased from 0.35 to 123.25 kJ m^−3^, showing *ε*_max_‐dependent energy dissipation behavior (Figure S4a,b and Table S2, Supporting Information). From a network microstructure viewpoint, PN‐BSA gels displayed irregular‐shape aggregate structures due to heat‐induced BSA denaturation (Figure 3d,e), although PC‐BSA gels had a highly porous structure with a wide pore size distribution from 0.5 to 4 µm (Figure 3f,g). PNC‐BSA gels displayed similar heterogeneous pore structures to PC‐BSA gels (Figure 3h,i), but the co‐presence of EDC/NHS‐crosslinked BSA and denatured BSA rendered the PNC‐BSA gel network much denser than that of PC‐BSA gels. Variation in morphologic properties also implies changes in the physical–chemical properties of these hydrogels. Regarding why PC‐BSA gel showed the higher strain than PN‐BSA gel and PNC‐BSA gel, in Figure 3d,e, since PC‐BSA gels were chemically‐crosslinked by amido groups while still retaining intact BSA proteins in association with PC network, the gels showed a fully continuous network with a dense porous structure, similar to other polymer hydrogels. Initial stretching of PC‐BSA gels caused the deformation of the entire network to dissipate energy, but further stretching will induce the intact BSA proteins to denature for the second‐time energy dissipation. Differently, PN‐BSA gels were physically‐crosslinked by heat‐induced, denatured BSA proteins, thus BSA network was not fully continuous with an irregular aggregate structure, which imposed the adverse effect on bear‐loading property, leading to their weak tensile property. Therefore, the PC‐BSA gels showed not only yielding behavior but also a higher strain than PN‐BSA gels. PNC‐BSA gels had similar pore structures to PC‐BSA gels, but with a much higher crosslinking density, which provided a much higher tensile strength than PC‐BSA. However, denatured BSA proteins in PNC‐BSA cannot provide additional deformation bearing property, thus leading to similar breaking‐strain to PN‐BSA gels, but much less strain than PC‐BSA gels.

To better understand the mechanical reinforcement mechanisms of PNC‐BSA gels, EDC/NHS concentration (*C*_EDC/NHS_), BSA concentration (*C*_BSA_), heating temperature (*T*), and heating time (*t*) were varied to examine the mechanical properties of PNC‐BSA gels. All of mechanical results were summarized in Figure S5 and Tables S3–S6, Supporting Information. First, as shown in Figure S5a,e and Table S3, Supporting Information, *C*_EDC/NHS_ influenced both the appearance and strength of PNC‐BSA gels. As *C*_EDC/NHS_ < 2 wt%, PNC‐BSA gels displayed a milky and opaque appearance, with weak compressive strength (*σ*_c,f_ = ≈4 MPa). As *C*_EDC/NHS_ ≥ 2 wt%, PNC‐BSA gels became light yellow and transparent, and its compressive strength gradually increased as *C*_EDC/NHS_ and achieved a maximal value of 115 MPa at 6 wt%. However, further increase of *C*_EDC/NHS_ to 10 wt% led to a decrease of *σ*_c,f_ to 26 MPa, indicating the over‐crosslinking effect of EDC/NHS. Only optimal *C*_EDC/NHS_ allows to form the compatible chemically‐linked BSA network with heat‐induced physical BSA network, thus achieving the high strength and transparence of PNC‐BSA gels. Low *C*_EDC/NHS_ of < 2 wt% cannot form the effective chemically‐crosslinked network, while high *C*_EDC/NHS_ often cause an over‐crosslinking issue. Second, PNC‐BSA gels improved their mechanical strength and optical transparence as *C*_BSA_ (Figure S5b,f and Table S4, Supporting Information), that is, PNC‐BSA gels prepared at *C*_BSA_ ≤ 200 mg mL^−1^ displayed milky and opaque and were too brittle to be tested, but as *C*_BSA_ ≥ 300 mg mL^−1^, PNC‐BSA gels became more transparency and its *σ*_c,f_ was increased from 4 to 115 MPa as *C*_BSA_ increased from 300 to 500 mg mL^−1^.

Due to heat‐induced denaturation of BSA proteins, heating temperatures and heating times are also important factors to affect the gelation process and subsequent mechanical properties of PNC‐BSA gels. It can be seen in Figure S5c and Table S5, Supporting Information that as *T* approached ≈50–70 °C, PNC‐BSA gels showed a relatively low *σ*_c,f_ (23–26 MPa), similar to that of PC‐BSA gels (20 MPa). Further increase of *T* to 80 °C led to a significant increase of *σ*_c,f_ to 115 MPa. Evidently, the higher *T* favors to facilitate the formation of denser denaturalized BSA network, which helps to improve the compression strength of PNC‐BSA gels. In addition, PNC‐BSA gels reached the best mechanical properties of *E*_c_ of 74–946 kPa, *ε*_c,f_ of 84.3–89.3%, and *σ*_c,f_ of 3–51 MPa at *t* = 10 min (Figure S5d and Table S6, Supporting Information). Unless otherwise stated, PNC‐BSA gels prepared at the optimal conditions (*C*_EDC/NHS_ = 6 wt%, *C*_BSA_ = 500 mg mL^−1^, *T* = 80 °C, *t* = 10 min) were used for subsequent studies.

Different from polymer‐based hydrogels amenable to swelling easily, PNC‐BSA gels were highly resistant to swelling. As shown in Figure S6, Supporting Information, PNC‐BSA gel enabled to retain its original shape when it was immersed into water for 7 days (i.e., swelling ratio was ≈1 in water), indicating that protein‐formed networks are not swellable in solution, simply because even denatured proteins do not swell due to their unique misfolding structures. However, PNC‐BSA gels underwent the swelling phenomenon in other organic solvents, as evidenced by swelling ratios of 1.53 in 4 m guanidine hydrochloride (GdnHCl), 2.5 in 8 m urea, and 2.18 in 20 mm SDS solution. As shown in **Table** **1**, upon swelling of PNC‐BSA gels in water, they can still retain the high compressive stress of 62.13 MPa, comparable to that of as‐prepared PNC‐BSA gels. The non‐swelling phenomenon of PNC‐BSA gels in water is likely due to the tightly unfolded BSA structures, which were not disrupted by water molecules. In contrast, after immersing PNC‐BSA gels in GdnHCl, urea, and SDS solutions, compressive strength of PNC‐BSA gels was significantly dropped to 0.1, 0.021, and 0.05 MPa, respectively. Such differences indicate that these solutions destabilize the unfolded structures of BSA, which reduces the associations and interactions between BSA proteins.

**Table 1:** Compressive properties of PNC‐BSA gels after swelling in various solutions

Gels (solvent)	*E*_c_ [kPa]	*ε*_c,f_ [%]	*σ*_c,f_ [MPa]
PNC‐BSA gel (a.p.)^a)^	971 ± 13	89.29 ± 0.11	115.6 ± 5.16
PNC‐BSA gel (water)	903 ± 7	89.14 ± 0.23	62.13 ± 3.67
PNC‐BSA gel (GdnHCl)	49 ± 4	54.91 ± 2.87	0.1 ± 0.014
PNC‐BSA gel (urea)	17 ± 1	46.14 ± 2.27	0.021 ± 0.0047
PNC‐BSA gel (SDS)	50 ± 4	44.24 ± 2.43	0.05 ± 0.0074

^a)^ a.p. means as‐prepared.

### 3.3 Self‐Recovery and Fatigue Resistance of PNC‐BSA Gels

**Figure 4:**
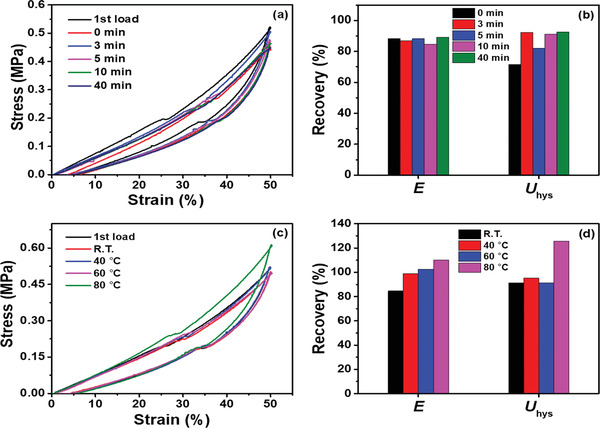
Self‐recovery of PNC‐BSA gels at different resting times and different temperatures. a) Cyclic loading‐unloading curves and b) mechanical recovery of PNC‐BSA gels at *ε*_max_ of 50%, room temperature, and different resting times of 0–40 min. c) Cyclic loading‐unloading curves and d) mechanical recovery of PNC‐BSA gels at *ε*_max_ of 50%, resting time of 10 min. and different heating times of 25–80 °C.

Due to the presence of physical interactions in BSA network, we examined the self‐recovery potential of PNC‐BSA gels using cyclic loading‐unloading tests. In **Figure** **4**a,b, PNC‐BSA gel displayed rapid self‐recovery even at room temperature. After the first loading‐unloading cycle, the gel can immediately recover 88% of stiffness and 72% of toughness without any resting time. Increase of resting times from 0 to 40 min, the gels significantly recovered their toughness from 72% to 92%, while retaining stiffness recovery to similar levels of 85–89%. Moreover, increase in heating temperature can simultaneously promote both stiffness and toughness recoveries. At 60 °C, the stiffness and toughness were recovered to 100% and 91%, respectively, which were much better than the recoveries at room temperature. Stiffness and toughness recoveries at 80 °C became more pronounced to achieve 110 and 126%, respectively, which is likely attributed to the secondary heat‐induced BSA denaturation (Figure 4c,d).

**Figure 5:**
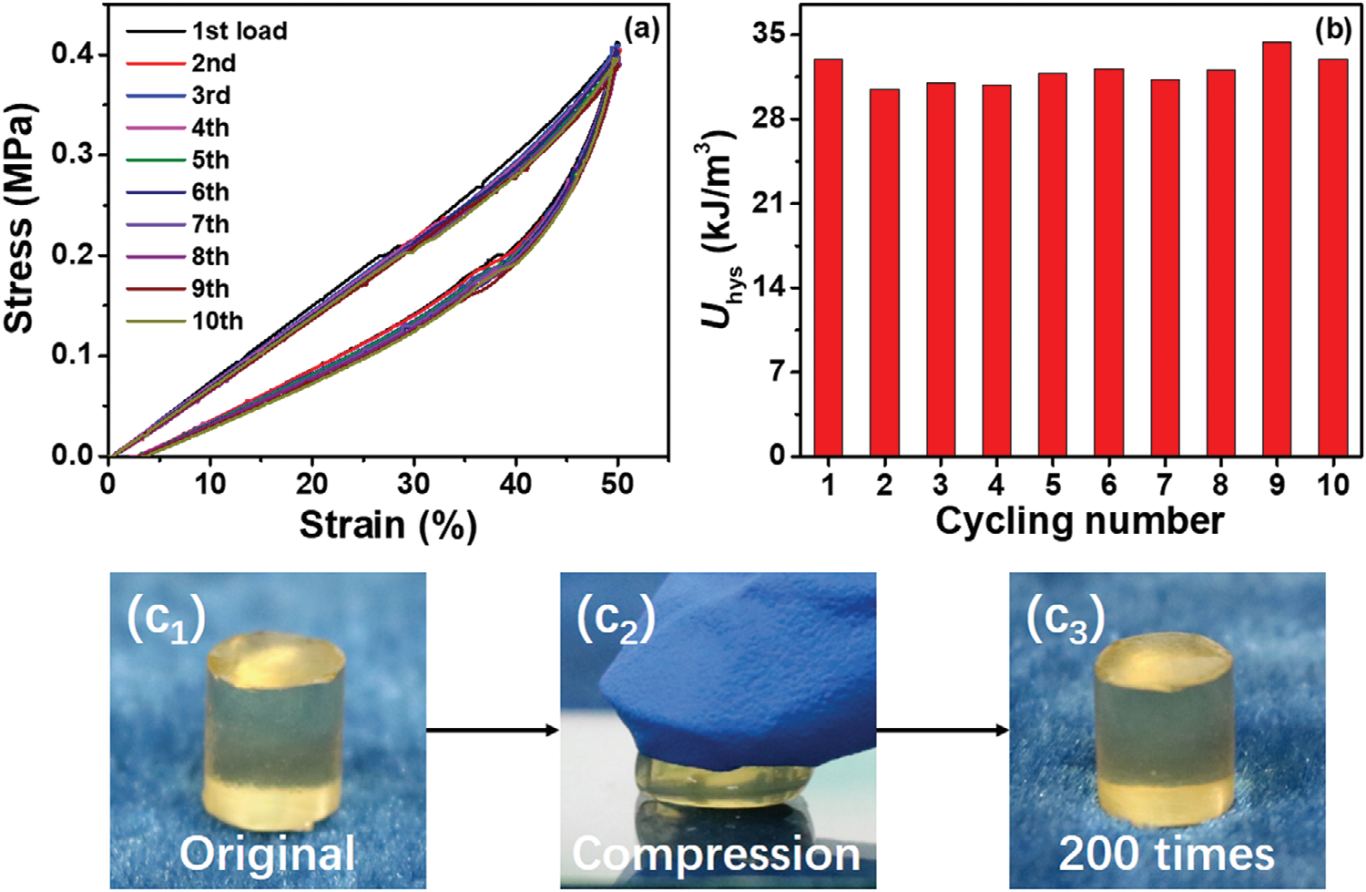
Fatigue resistant property of PNC‐BSA gels. a) 10 successive loading‐unloading curves and b) the corresponding dissipated energies of PNC‐BSA gels. During the tests, resting time of 5 min was applied to the gel between two consecutive loadings. c) Visual inspection of PNC‐BSA gels before, during, and after 200‐times compression.

Hydrogels, regardless of their stiffness and dissipation levels, do not necessarily present a fatigue resistance capability. We applied 10 successive loading cycles to assess the fatigue resistance of PNC‐BSA gels with 5 min resting time between cyclic loadings at room temperature. As shown in **Figure** **5**a, all of 10 hysteresis loops were almost completely overlapped with each other. Consistently, the dissipated energies remained almost the same for each loading cycle (31–34 kJ m^−3^) (Figure 5b). Further, after 200‐time compression on the same gel, we did not visually observe any apparent damage. These data indicate that PNC‐BSA gels can well retain their network structures after many repeated mechanical deformations, demonstrating excellent fatigue resistance.

### 3.4 Comparison of PNC‐BSA Gels with Other Natural Protein‐Based Gels

To prove that our fabrication method could be generally applicable to other protein‐based hydrogels via the PNC strategy, we used the same fabrication method to prepare different PNC‐protein hydrogels using silk fibrin (SF), gelatin, ovalbumin (OVA), and milk albumin (MA) proteins, where the former two proteins are typical fibrin proteins, while the latter two are typical globulin proteins. As shown in **Table** **2**, upon introducing protein denaturization and EDC/NHS, all PNC‐protein gels exhibited the high compressive properties, as evidenced by compressive strength of 0.33–4.56 MPa. As expected, PN‐SF gels were too weak to be handled. In sharp contrast, upon introducing EDC/NHS crosslinking, PNC‐SF gel can achieve *E*_c_ of 979 kPa and *σ*_c,f_ of 0.94 MPa. Similarly, gelatin as a thermo‐reversible biopolymer can be gelled below melting point (*T*_m_). PNC‐gelatin gels displayed *σ*_c,f_ of 0.33 MPa. It is likely that EDC/NHS‐crosslinked network hinders the aggregation of triple helix bundles to form a physically crosslinked network, leading to the less improved mechanical property. Different from SF and gelatin but similar to BSA, OVA, and MA can be readily gelled upon heating. Impressively, both PNC‐OVA and PNC‐MA gels exhibited high *σ* values of 1.66 and 4.56 MPa, respectively, while both gels possessed the lower compressive properties than PNC‐BSA gels. We also listed some protein hydrogels created by other strategies and their mechanical properties in Table 2, which showed that our PNC strategy had better mechanical properties in gelatin, OVA, and MA based hydrogels. Upon examination of different protein hydrogel systems in terms of their gelation process and mechanical properties, we demonstrated a new, general strategy, combining heat‐induced unfolding and EDC/NHS coupling, to fabricate different PNC‐protein hydrogels with high strength.

**Table 2:** Mechanical properties of different protein hydrogels

Gels	*E*_c_ [kPa]	*ε*_c,f_ [%]	*σ*_c,f_ [MPa]	Ref.
PNC‐BSA gel	971 ± 13	89.29 ± 0.11	115.6 ± 5.16	This work
PNC‐SF gel	979 ± 162	67.02 ± 3.12	0.94 ± 0.12	This work
PNC‐Gelatin gel	84 ± 19	68.10 ± 1.81	0.33 ± 0.067	This work
PNC‐OVA gel	561 ± 3	72.79 ± 2.97	1.66 ± 0.21	This work
PNC‐MA gel	68 ± 2	88.35 ± 0.47	4.56 ± 0.38	This work
HRP/H_2_O_2_‐SF gel^a)^	3000	60	1.7	^[^ ^51^ ^]^
PC‐Gelatin gel^b)^	4	40	5 × 10^−3^	^[^ ^44^ ^]^
SPFO‐OVA gel^c)^	0.2–0.3	/	/	^[^ ^52^ ^]^
HRP/H_2_O_2_‐MA gel^d)^	0.2–0.4	/	/	^[^ ^53^ ^]^

^a)^ HRP means horseradish peroxide, SF concentration was 10 wt%, the gel did not break under 60% strain

^b)^ Gelatin concentration was 5% w/v, mass ratio of EDC/NHS:gelatin was 1:12

^c)^ SPFO means sodium perfluorooctanoate, OVA concentration was 40 mg mL^−1^

^d)^ MA concentration was 5.6% w/v, mass ratio of MA:HRP was 20:1. (Modulus in c and d were G′ obtained from rheological tests.)

### 3.5 Potential Applications

**Figure 6:**
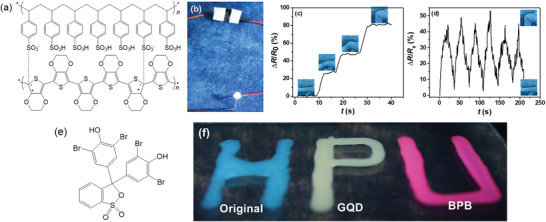
Functionalization of PNC‐BSA gels. a) Chemical structure of PEDOT/PSS, b–d) Electrical performance of PEDOT/PSS‐incorporated PNC‐BSA gels; e) Chemical structure of bromophenol blue (BPB), f) PNC‐BSA gels with and without graphene quantum dot (GQD) and BPB to emit different colors under UV radiation.

We further applied PNC‐BSA gels to be used as smart sensors. Our simple, one‐pot method allows to readily incorporate nanoparticles into PNC‐BSA gels for achieving additional functions. As a proof‐of‐concept example, we introduced conductive PEDOT/PSS nanoparticles into PNC‐BSA gels to form PEDOT/PSS@PNC‐BSA gel (**Figure** **6**a). The conductive PEDOT/PSS components enable PEDOT/PSS@PNC‐BSA gels to be used as not only a conductor to lighten a LED light (Figure 6b), but also as a strain senor to monitor the human motions. When attaching PEDOT/PSS@PNC‐BSA gel to finger, bending of the finger from 0° to 90° allows to immediately detect the relative resistance change (Figure 6c). Remain of bending status did not induce any change in relative resistances, indicating a good electrical signal stability. Next, multiple bending of the finger back‐and‐forth between bent (90°) and straighten (0°) status resulted in the reversible signal changes of relative resistance with similar signal intensity, indicating stable sensing repeatability (Figure 6d). In Figure S7, Supporting Information, conductive 2D MXene nanoplates were also added into the precursor solution to fabricate Mxene@PNC‐BSA gels. Resultant PNC‐BSA/Mxene gels can also be used as strain sensors to monitor the human motions. The results indicate that the introduction of nanofillers into PNC‐BSA gel is a general way to empower different functions of our protein gels. Therefore, GQD and BPB (Figure 6e) were added separately into PNC‐BSA gels, leading to GQD@PNC‐BSA and BPB@PNC‐BSA gels. Interestingly, under 365 nm UV light, original PNC‐BSA, GQD@PNC‐BSA, and BPB@PNC‐BSA gels emitted blue, yellow, and purple fluorescence, respectively, as clearly visualized by blue “H,” yellow “P,” and purple “U” letters (Figure 6f, HPU stands for Henan Polytechnic University).

Biocompatibility of hydrogels is another important property for many bio‐related applications. Since BSA as a common protein is often used in cell culture, purely as‐prepared BSA hydrogels are expected to be naturally biocompatible with cells. Herein, we conducted cell cytotoxicity and hemolysis tests to investigate the biocompatibility of PNC‐BSA gels. As shown in Figure S8a, Supporting Information, when leach liquor of PNC‐BSA gels was incubated with L929 cells for 24 h, PNC‐BSA gels did not appear to have any significant deleterious effect on cell viability. Instead, PNC‐BSA gels induced 45% higher cell viability than control samples (cell alone) and all living cells also retained their healthy morphology. This indicates that PNC‐BSA gels promote the cell proliferation, which is probably attributed to both effects of high biocompatibility of PNC‐BSA gels and 3D network structure of hydrogels being an adequate environment for living cells. In parallel, for any biocompatible materials, hemolysis of RBCs is equally important to cell viability. For comparison, PNC‐BSA gels, negative control, and positive control were used under the same conditions. As shown in Figure S8b, Supporting Information, after 1‐day storage, the hemolysis ratio of preserved RBCs was only 4% in a bag PNC‐BSA gels, as compared to the positive control. Additionally, most RBCs in the PNC‐BSA gels exhibited biconcave shape, further serving as an indicator that the RBCs are capable of remaining viable in PNC‐BSA cultures. As shown in Figure S9, Supporting Information, using porcine skin as model, PC‐BSA gel can be used as a tissue adhesive for potential wound dressings. Accordingly, the adhesive properties of PC‐BSA gels to porcine skin were evaluated by 180° peeling (Figure S9a, Supporting Information) and lap shear tests (Figure S9b, Supporting Information). The peeling curves of PC‐BSA gels from the porcine skin showed adhesive energy of 394 ± 16 J m^−2^ (Figure S9c, Supporting Information) and adhesive strength of 134 ± 18 kPa (Figure S9d, Supporting Information). The results indicate that PC‐BSA gels can toughly and strongly adhere to soft tissues for tissue adhesive applications.

## 4 Conclusions

In this work, we proposed and demonstrated a general, thermal‐induced protein unfolding and EDC/NHS coupling reaction to prepare pure PNC‐protein hydrogels without any synthetic polymer. Different from other hybrid protein‐polymer hydrogels, the pure PNC‐BSA hydrogels, solely containing BSA proteins, were constructed to achieve both high mechanical property and excellent cell biocompatibility. Due to chemical‐crosslinking networks and misfolded protein structures, PNC‐BSA hydrogels showed high compressive strength of ≈115 MPa, fast stiffness/toughness recovery of 85%/91% after resting for 30 min at room temperature, high resistance to swelling, and excellent fatigue resistance without losing energy dissipation capacity after 10 cycles. Moreover, these PNC‐BSA hydrogels can effectively promote cell proliferation and reduce RBC hemolysis. From a practical viewpoint, PNC‐BSA hydrogels can also be functionalized with conductive PEDOT/PSS or MXene to realize strain sensors and with different fluorescents to emit different colors. This work provides a simple PNC strategy to prepare and strengthen pure protein hydrogels, which will greatly expand the high strength hydrogel families and the protein hydrogel applications.

## Conflict of Interest

The authors declare no conflict of interest.

## Supporting information

Supporting Information

## Data Availability

The data that support the findings of this study are available from the corresponding author upon reasonable request.
