# Supplementary material for: A General Protein Unfolding‐Chemical Coupling Strategy for Pure Protein Hydrogels with Mechanically Strong and Multifunctional Properties
Source: Adv Sci (Weinh). 2021 Dec 22;9(5):2102557. doi: 10.1002/advs.202102557 (PMC8844490; doi:10.1002/advs.202102557)
Supplement: Supplementary file 1 — Supporting Information [file ADVS-9-2102557-s001.pdf]

© 2021 Wiley-VCH GmbH

ADVANCED SCIENCE
Open Access

Supporting Information

for *Adv. Sci.*, DOI: 10.1002/advs.202102557

A General Protein Unfolding-Chemical Coupling Strategy for Pure Protein Hydrogels with Mechanically Strong and Multifunctional Properties

Ziqing Tang[1, #], Huacheng He[2, #], Lin Zhu[3], Zhuangzhuang Liu[4], Jia Yang[4], Gang Qin[4], Jiang Wu[5], Yijing Tang[6], Dong Zhang[6], Qiang Chen[1, 3]*, Jie Zheng[6]*

Supporting Information

A General Protein Unfolding-Chemical Coupling Strategy for Pure Protein Hydrogels with Mechanically Strong and Multifunctional Properties

Ziqing Tang[1, #], Huacheng He[2, #], Lin Zhu[3], Zhuangzhuang Liu[4], Jia Yang[4], Gang Qin[4], Jiang Wu[5], Yijing Tang[6], Dong Zhang[6], Qiang Chen[1, 3]*, Jie Zheng[6]*

[1]Wenzhou Institute, University of Chinese Academy of Sciences, Wenzhou, 325001, China.

[2]College of Chemistry and Materials Engineering, Wenzhou University, Wenzhou, Zhejiang 325035, China

[3] Oujiang Laboratory (Zhejiang Lab for Regenerative Medicine, Vision and Brain Health), Wenzhou, Zhejiang, China, 325000.

[4]School of Materials Science and Engineering, Henan Polytechnic University, Jiaozuo, 454003, China.

[5]School of Pharmaceutical Sciences, Key Laboratory of Biotechnology and Pharmaceutical Engineering, Wenzhou Medical University, Wenzhou, Zhejiang 325035, China

[6]Department of Chemical, Biomolecular, and Corrosion Engineering, The University of Akron, Akron, Ohio, 44325, USA.

[#] The authors contribute equally to this work.

*Corresponding author: chenqiang@ucas.ac.cn; zhengj@uakron.edu

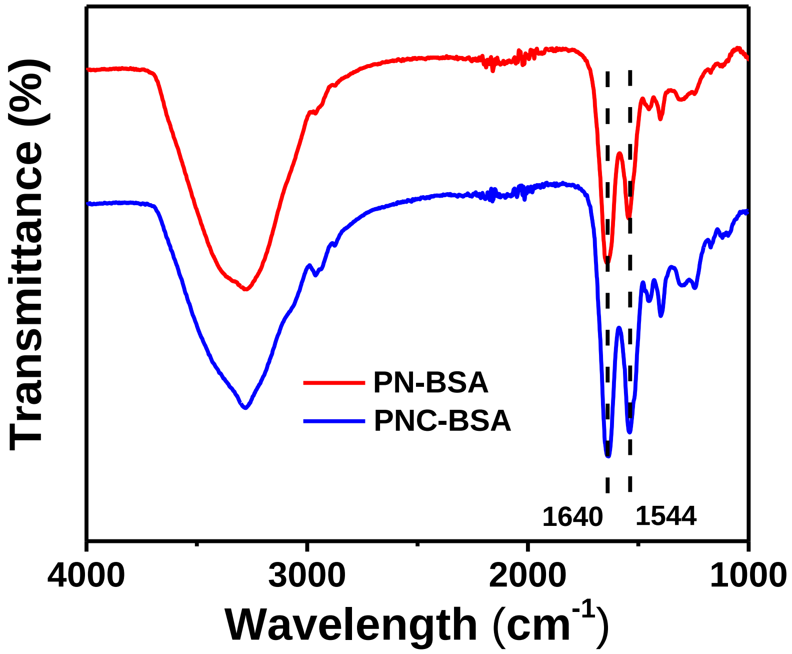

Figure S1. FTIR spectra of PN-BSA and PNC-BSA gels.

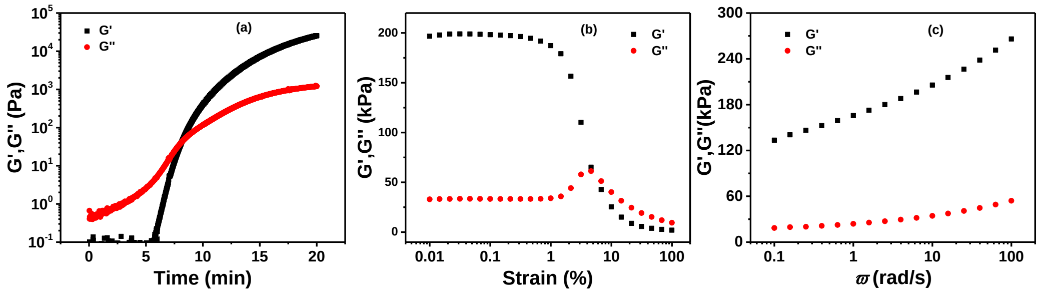

Figure S2. (a) Gelation time of PC-BSA gel, (b) amplitude sweep and (c) frequency sweep at 25 ℃ of PNC-BSA gel.

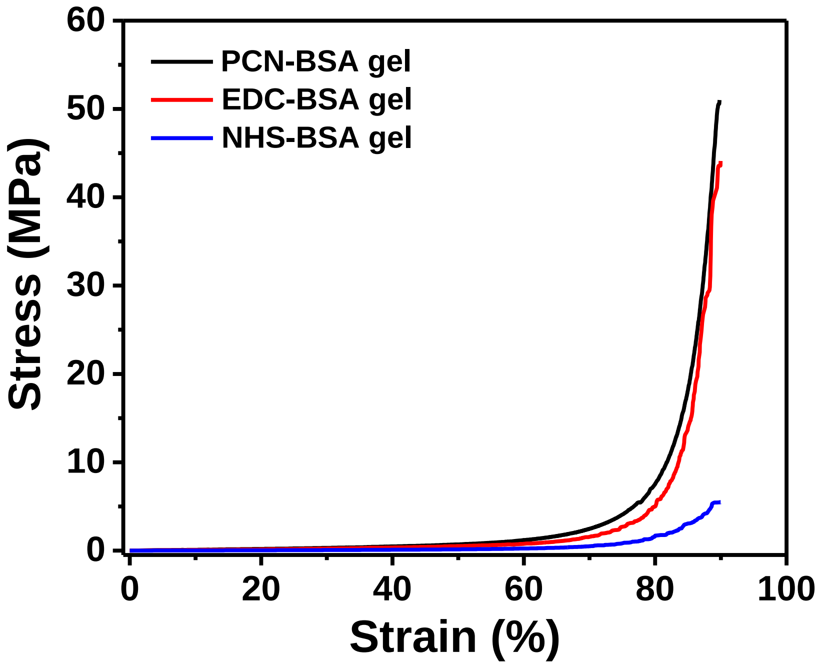

Figure S3. Compression curves of PCN-BSA gel, EDC-BSA gel and NHS-BSA gel.

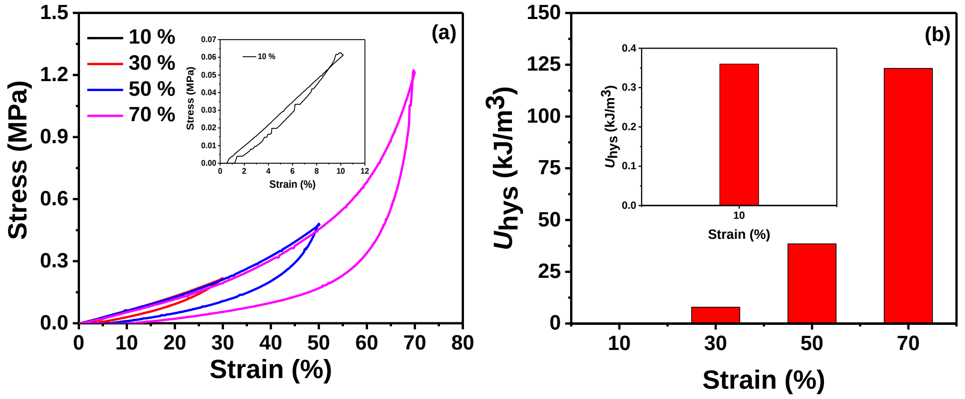

Figure S4. Loading-unloading curves and (b) U_{hys} of PNC-BSA gel at different compression ratios.

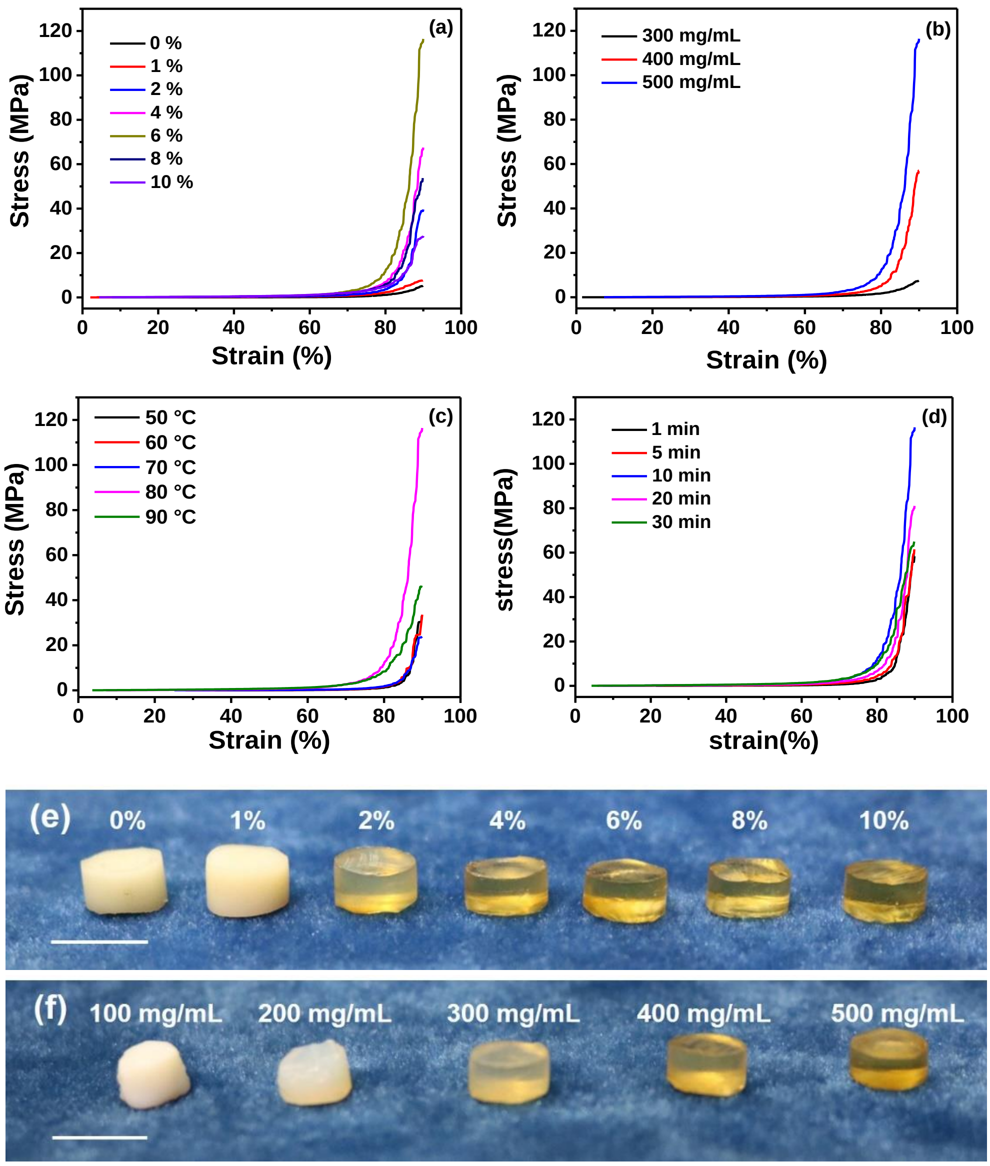

Figure S5. Effects of (a) EDC/NHS concentration, (b) BSA concentration, (c) heating time, and (d) heating temperature on the tensile properties of PNC-BSA gel. Photographs of PNC-BSA gels at different (e) $C_{\mathrm{EDC/NHS}}$ and (f) C_{BSA} (The scale bars in figures mean 1 cm).

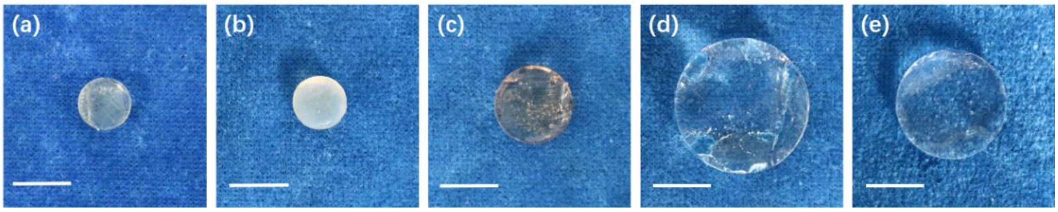

Figure S6. Photos of PNC-BSA gels after swelling in different solutes for 7 days: (a) original, (b) water, (c) 4 M GdnHCl, (d) 8 M Urea and (e) 20 mM SDS. (Scale bars mean 1 cm)

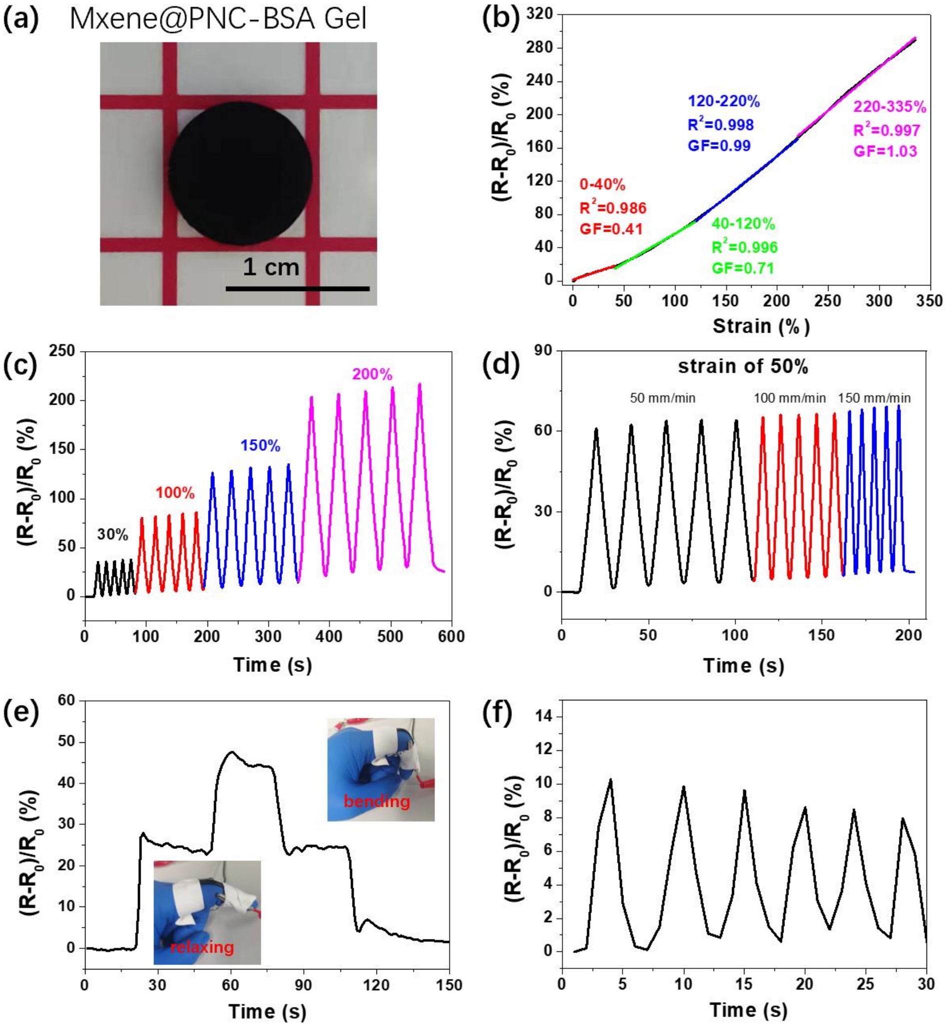

Figure S7. MXene@PNC-BSA hydrogel strain sensor. (a) Optical photograph of MXene@PNC-BSA hydrogel; (b) Gauge factor of MXene@PNC-BSA hydrogel strain sensor. Red, green, blue, and pink labels represent the fitting lines for tensile strains ranging from 0% to 40% (with linearity of 0.986), 40% to 120% (with linearity of 0.996), 120% to 220% (with linearity of 0.998), and 220% to 335% (with linearity of 0.997), respectively; (c) Relative resistance changes of MXene@PNC-BSA hydrogel strain sensor under various cyclic maximum strains (30%, 100%, 150%, 200%); (d) Relative resistance variation of MXene@PNC-BSA hydrogel strain sensor under different strain rates (50mm/min, 100mm/min, and 150mm/min) between 0-50% strain; Relative resistance variation of MXene@PNC-BSA hydrogel adhered onto the index finger to detect (e) different bendings and (f) repeated bendings of the finger.

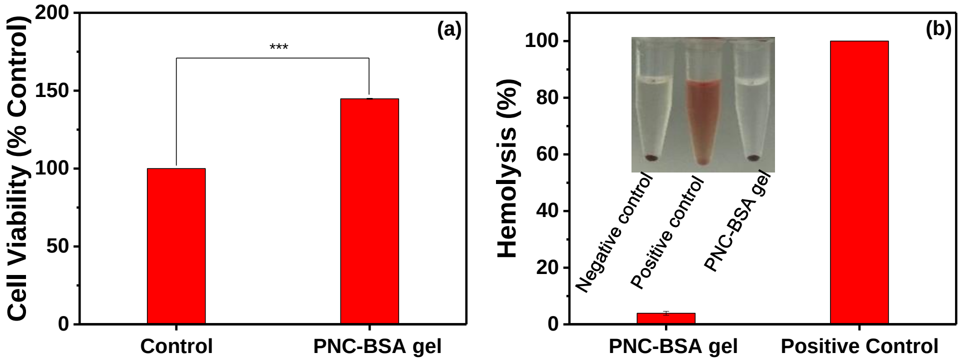

Figure S8. (a) Cytotoxicity and (b) hemolysis of PNC-BSA gels. N=4; *** shows the significant differences (Unpaired *t* test: *** $p < 0.001$).

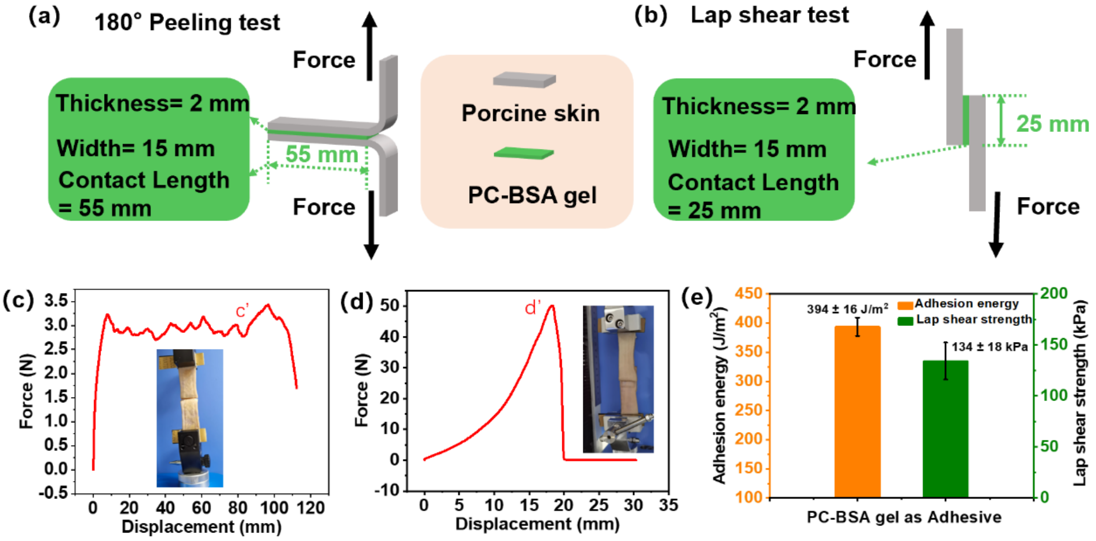

Figure S9. PC-BSA hydrogel as tissue adhesive. Scheme of (a) 180 ^{o} peeling test and (b) lap shear test for PC-BSA hydrogels on porcine skins. (c) Peeling force per width of hydrogel versus displacement for PC-BSA hydrogels using 180 ^{o} peeling test (Inset: steady state at c' point); (d) Peeling force per width of hydrogel versus displacement for PC-BSA hydrogels using lap shear test (Inset: a state before fracture at d' point); (e) Adhesion energy from peeling tests and lap shear strength from lap shear tests.

Table S1. Compression properties of various BSA gels

Gel samples	$\varepsilon_{c,f}$ (%)	$\sigma_{c,f}$ (Mpa)	E_c (kPa)
PNC-BSA gel	89.29±0.11	115.36±5.16	971±73
PN-BSA gel	88.91±0.58	4.21±0.36	74±2
PC-BSA gel	89.23±0.1	20.18±0.26	436±18
PCN-BSA gel	89.24±0.55	51.38±3.75	976±24
EDC-BSA gel	89.3±0.22	43.04±1.82	894±23
NHS-BSA gel	84.25±1.74	3.09±0.69	206±16

Table S2. U_{hys} of PNC-BSA gel at different compression ratios

Strain (%)	U_{hys} (kJ/m^3)
10	0.36
30	7.88
50	38.47
70	123.25

Table S3. Effect of $C_{EDC/NHS}$ on compression properties of PNC-BSA gel

EDC/NHS Concentration (wt%)	$\varepsilon_{c,f}$ (%)	$\sigma_{c,f}$ (MPa)	E_c (kPa)
0	88.91±0.58	4.21±0.36	74±2
1	84.09±0.90	3.94±0.33	345±8
2	89.37±0.11	39.24±5.93	641±84
4	89.12±0.20	58.16±3.10	1026±33
6	89.29±0.11	115.36±5.16	971±73
8	89.01±0.19	45.80±4.81	943±28
10	88.91±0.52	25.92±3.48	1012±42

Table S4. Effect of C_{BSA} on compression properties of PNC-BSA gel

BSA Concentration (mg/mL)	$\varepsilon_{c,f}$ (%)	$\sigma_{c,f}$ (MPa)	E_c (kPa)
300	85.31±0.92	3.90±0.45	214±19
400	89.31±0.21	55.91±3.61	641±10
500	89.29±0.11	115.36±5.16	971±73

Table S5. Effect of heating temperatures on compression properties of PNC-BSA gel

T (℃)	$\varepsilon_{c,f}$ (%)	$\sigma_{c,f}$ (MPa)	E_c (kPa)
50	89.42±0.4	26.15±5.85	361±51
60	88.8±0.62	23.36±1.64	304±10
70	88.98±0.27	23.48±2.33	377±20
80	89.29±0.11	115.36±5.16	971±73
90	89±0.22	41.25±1.67	1351±15

Table S6. Effect of heating times on compression properties of PNC-BSA gel

t (min)	$\varepsilon_{c,f}$ (%)	$\sigma_{c,f}$ (MPa)	E_c (kPa)
1	89.36±0.07	53.15±3.36	320±30
5	89.32±0.20	60.1±2.84	566±20
10	89.29±0.11	115.36±5.16	971±73
20	89.42±0.20	76.56±7.00	862±7
30	88.82±0.27	60.44±6.72	1359±23
